# Distinctive alterations in the functional anatomy of the cerebral cortex in pain-sensitized osteoarthritis and fibromyalgia patients

**DOI:** 10.1186/s13075-022-02942-3

**Published:** 2022-11-11

**Authors:** Jesus Pujol, Laura Blanco-Hinojo, Andrea Doreste, Fabiola Ojeda, Gerard Martínez-Vilavella, Víctor Pérez-Sola, Joan Deus, Jordi Monfort

**Affiliations:** 1 MRI Research Unit, Department of Radiology, Hospital del Mar, Passeig Marítim 25-29. , 08003 Barcelona, Spain; 2 CIBER de Salud Mental, Instituto de Salud Carlos III, Barcelona, Spain; 3 Rheumatology Department, Hospital del Mar, Barcelona, Spain; 4 Institute of Neuropsychiatry and Addictions, Hospital del Mar- IMIM, Pompeu i Fabra University, Barcelona, Spain; 5 Department of Psychobiology and Methodology in Health Sciences, Autonomous University of Barcelona, Barcelona, Spain

**Keywords:** Functional MRI, Pain sensitization, Fibromyalgia, Knee osteoarthritis, Somatosensory cortex, Insular cortex

## Abstract

**Background:**

Pain-sensitized osteoarthritis and fibromyalgia patients characteristically show nociceptive system augmented responsiveness as a common feature. However, sensitization can be originally related to the peripheral injury in osteoarthritis patients, whereas pain and bodily discomfort spontaneously occur in fibromyalgia with no apparent origin. We investigated the distinct functional repercussion of pain sensitization in the cerebral cortex in both conditions.

**Methods:**

Thirty-one pain-sensitized knee osteoarthritis patients and 38 fibromyalgia patients were compared with matched control groups. And new samples of 34 sensitized knee osteoarthritis and 63 fibromyalgia patients were used to directly compare each condition. A combined measure of local functional connectivity was estimated to map functional alterations in the cerebral cortex at rest.

**Results:**

In osteoarthritis, weaker local connectivity was identified in the insula, which is a cortical area processing important aspects of the brain response to painful stimulation. In contrast, fibromyalgia patients showed weaker connectivity in the sensorimotor cortex extensively affecting the cortical representation of the body.

**Conclusions:**

In osteoarthritis, weaker insular cortex connectivity is compatible with reduced neural activity during metabolic recovery after repeated activation. In the fibromyalgia neurophysiological context, weaker connectivity may better express both reduced neural activity and increased excitability, particularly affecting the sensorimotor cortex in patients with spontaneous body pain. Such a combination is compatible with a central gain enhancement mechanism, where low sensory tolerance results from the over-amplification of central sensory reception to compensate a presumably weak sensory input. We propose that deficient proprioception could be a factor contributing to weak sensory input.

**Supplementary Information:**

The online version contains supplementary material available at 10.1186/s13075-022-02942-3.

## Background

Knee osteoarthritis is often associated with augmented pain responses due to the sensitization of the nociceptive system [1, 2]. In this case, lower pain thresholds and regional spreading of pain can be originally related to the peripheral damage. In contrast, augmented pain sensitivity characteristically occurs in fibromyalgia with no evident origin [3]. The International Association for the Study of Pain (IASP) has recently proposed the term nociplastic pain precisely to indicate the presence of pain arising from altered nociception despite no clear evidence of tissue damage or lesion of the somatosensory system [4, 5].

A number of changes in functional connectivity have been identified on the brain scale in knee osteoarthritis [6–11]. The insula appears to be particularly affected with a loss of its central role as a hub in pain network organization [8] and altered functional relationships with other networks [7, 8, 11]. In fibromyalgia, changes in connectivity have been identified in the insula (e.g., [12]) and each nociception-processing level (e.g., [2, 13–16]), including the sensorimotor cortex [2, 14, 15] and affecting areas processing other sensory modalities [2, 14]. Interestingly, the insula may reinforce its role as a connectivity hub [14] and the coupling to the posterior cingulate cortex [16] in fibromyalgia in the opposite direction to the effect observed in knee osteoarthritis [7, 8]. All in all, the available data at the large cortical scale led us to hypothesize that alterations in cerebral cortex local connectivity measures would be distinct in primary and secondary sensitization disorders.

We investigated the functional repercussions of pain sensitization on cerebral cortex local, short-distance connectivity using iso-distance average correlation (IDAC) measures [17]. IDAC expresses the functional coupling of a brain unit (voxel) with their neighboring elements. Three IDAC measures were combined to inform on the rich spatial structure of cortical connectivity. Local functional connectivity maps may provide information about cortical functioning insofar as variations in local connectivity are related to neural activity variations. However, for optimal interpretation, it is important to consider that variations in local connectivity are related to variations in the activity of both principal/pyramidal neurons and inhibitory interneurons. That is, stronger connectivity may express higher principal neuron activity (more coactivated elements) [17], but also higher inhibitory interneuron activity (higher neural synchronization) [18], and vice versa [19].

In this study, we used combined IDAC measures to identify changes in local functional connectivity of the cerebral cortex in pain-sensitized knee osteoarthritis and fibromyalgia patients. Each clinical condition was primarily compared with a matched control group and new samples were used to directly compare both disorders. We suspected that the functional anatomy of the cerebral cortex may be affected differently in primary and secondary sensitization disorders to the extent that sensitization could have different origins. In such a context, the identification of distinctive alterations may contribute to a better understanding of the sensitization phenomena.

## Methods

All studies were conducted in accordance with the principles expressed in the Declaration of Helsinki, and each protocol was approved by the Ethical Committee of Clinical Research of the Parc de Salut Mar of Barcelona (ref. MP-TAP-2016–01) or the Ethics and Institutional Review Board of the Autonomous University of Barcelona (ref. PID2021-127703OB-I00). All participants provided written informed consent.

### Study design

This was an observational case–control study that used historical samples to compare each patient’s condition in terms of the IDAC measures with a corresponding control group matched by age and sex. And new patient samples were recruited to directly compare both disorders.

### Study populations

The study was conducted at the Hospital del Mar, Barcelona, Spain. All the participants were recruited by Rheumatology researchers from the Osteoarthritis and Fibromyalgia Referral Units. Historical samples included 31 pain-sensitized knee osteoarthritis patients (Osteoarthritis Sample 1) matched to 23 control subjects [2] and 38 fibromyalgia patients (Fibromyalgia Sample 1) to 35 control subjects [20]. The new samples included 34 sensitized knee osteoarthritis patients (Osteoarthritis Sample 2) recruited in 2019 and 2020 and 63 fibromyalgia patients (Fibromyalgia Sample 2) recruited in 2021 and 2022. Patients from the new samples participated in two different intervention studies (EudraCT registration 2016–005,082-31 and ClinicalTrials.gov identifier NCT03785535). Only baseline (prior to any intervention) images were used in the present analysis.

Sample selection and inclusion criteria have been previously described [2, 20, 21]. In short, the included osteoarthritis patients showed a radiological and clinical diagnosis of knee osteoarthritis based upon the American Rheumatism Association criteria [22] and pain sensitization affecting the knee defined by combining clinical and experimental (pain thresholds) evidence (see below) [2, 21]. Fibromyalgia patients were all diagnosed by a rheumatologist in accordance with the American College of Rheumatology criteria for fibromyalgia [23] and were consecutively recruited during clinical follow-up to make up a homogeneous sample with durable and severe symptoms (minimum Fibromyalgia Impact Questionnaire (FIQ) score [24], 40 out of 100 points). As for the control groups, subjects with relevant medical or neurological disorder, any form of chronic or acute pain, substance abuse, or psychiatric disease were not considered for inclusion.

### Clinical assessments

#### Osteoarthritis samples

Screening included complete medical history, physical examination, ECG, and X-ray of the knee. The presence and severity of pain sensitization were evaluated by expert clinical judgment, pain intensity scores in the preceding 24 h based on items 3 to 6 of the Brief Pain Inventory [25], number of tender points around the knee on the 10-site extended Arendt-Nielsen peripatellar map [1, 2], and pain temporal summation in an 11-point numerical rating scale (NRS) after 10 repeated pressure stimulations on the anterior/medial tibial surface [2]. Additional clinical assessments included The Western Ontario and McMaster Universities Osteoarthritis Index (WOMAC) physical-function scale (Cronbach’s alpha internal consistency ranging from 0.70 to 0.95) [26], the PainDETECT questionnaire (Cronbach’s alpha of 0.86 for the whole scale) [27], and an 11-point pain rating generated with a pressure of 4 kg/cm^2^ during 2 s on the tibial surface.

#### Fibromyalgia samples

Clinical assessments included complete medical history and physical examination, review of the American College of Rheumatology criteria for fibromyalgia [23], number of tender points out of 18 body sites (pain threshold equal or under 4 kg/cm^2^), Fibromyalgia Impact Questionnaire (Cronbach’s alpha of 0.82 for the total items) [24], General Perception of Health according to the 36-Item Short-Form Health Survey (Cronbach’s alpha > 0.75) [28, 29], Hospital Anxiety and Depression Scale (HADS) ratings (Cronbach’s alpha of 0.86 for both Anxiety and Depression sub-scales) [30], and a 101-point numerical rating of spontaneous (non-evoked) pain globally perceived in the body before functional magnetic resonance imaging (MRI).

### MRI acquisition

In all study samples, functional MRI was acquired in the resting state and as the first sequence in the session. Participants were instructed to relax, stay awake, and lie still, while keeping their eyes closed throughout. Each scan was obtained using eight-channel phased-array head coils and single-shot echo-planar imaging (EPI) software. The functional sequences always consisted of gradient recalled acquisition in the steady state, lasted 6 min, and generated 180 whole-brain image volumes (TR, 2 s).

Functional MRI for the Osteoarthritis Sample 1 and the corresponding control group was acquired using a 1.5-T Signa Excite system (General Electric, Milwaukee, WI, USA) with acquisition parameters set as repetition time, 2000 ms; echo time, 50 ms; pulse angle, 90°; 24-cm field of view; 64 × 64-pixel matrix; and slice thickness 4 mm. Functional MRI for the rest of the samples was acquired using a Philips Achieva 3.0-T magnet (Philips Healthcare, Best, The Netherlands) with repetition time, 2000 ms; echo time, 35 ms; pulse angle, 70°; 23-cm field of view; 64 × 64-pixel matrix; and slice thickness, 3.59 mm. High-resolution 3D anatomical images were also obtained in each case based on a T1-weighted three-dimensional fast spoiled gradient inversion recovery-prepared sequence, which served to assist image processing.

### Iso-distant average correlation (IDAC) maps

Imaging data were processed using MATLAB version 2016a (The MathWorks Inc, Natick, Mass) and Statistical Parametric Mapping software (SPM12; The Wellcome Department of Imaging Neuroscience, London). Image processing steps adopted to generate the cerebral cortex IDAC maps have been previously reported [17], and a detailed description is provided in Additional file 1. Below is a summary.

Functional MRI images were slice-time corrected, realigned, co-registered to their corresponding anatomical image, re-sliced to 3 × 3 × 3 mm resolution, and smoothed by convolving the image with a 4 × 4 × 4 mm full width at half maximum (FWHM) Gaussian kernel. Motion-affected image volumes were discarded using conventional scrubbing procedures [31].

IDAC measures were then estimated in native space. The computation was conducted in a gray matter mask split into left and right hemispheres. Whole-cortex IDAC maps were generated by estimating the average temporal correlation of each voxel with all its neighboring voxels placed at increasingly separated Euclidean iso-distant intervals (definition and mathematical formulation are provided in the Additional file 1). Three IDAC maps were obtained at distance intervals of 5–10 mm, 15–20 mm, and 25–30 mm. The analyses were adjusted by including 6 rigid body realignment parameters, their first-order derivatives, average white matter, CSF, and global brain signal as regressors. All functional MRI time series were band-passed with a discrete cosine transform (DCT) filter letting through frequencies in the 0.01–0.1-Hz interval.

Finally, the resulting IDAC maps in native space were normalized to the Montreal Neurological Institute (MNI) space with a back-transformation process. That is, original EPI images had previously been normalized using study-specific templates based on the 3D anatomical images and the estimated parameters were applied to the inverse normalization of IDAC maps.

Multi-distance IDAC color maps were obtained from the overlay of the three IDAC maps using an RGB color codification (see Figs. 2 and 4). RGB color channels enabled the display of three values simultaneously. Red corresponds to the results from 5–10-mm IDAC map analyses, green from 15–20 mm, and blue from 25–30 mm. The overlapping of these primary colors produces a full range of secondary colors. Composite RGB maps were generated from the between-group comparison t-maps obtained for each distance.

### Statistical analysis

#### Sample size

There is no consensus as to sample size estimation for functional MRI studies. Empirical experiments have demonstrated that a number above 24 subjects is optimal to achieve 80% power for a corrected threshold of 0.05 in conventional task activation experiments [32].

We used a double approach to characterize cerebral cortex functional alterations in two patient conditions representing distinct age populations. Firstly, the comparison of each clinical disorder with a matched control group allowed us to appreciate qualitative differences as to the functional anatomy of the alterations. In a second analysis, new samples of osteoarthritis and fibromyalgia patients were directly compared using age as a covariate which enabled us to confirm group differences in the cortical distribution of changes. In addition, the age effect on connectivity measures was directly assessed with a correlation analysis to further separate age from clinical condition effects.

IDAC connectivity maps were included in SPM group-wise random-effects analyses adopting a 2 × 3 mixed design ANOVA (ANCOVA) model (e.g., group [patients, controls] by distance [5–10 mm, 15–20 mm, and 25–30 mm]). A motion summary measure (mean inter-frame motion [31]) for each participant was included as a covariate in all analyses and age when directly comparing osteoarthritis with fibromyalgia patients. We specifically tested for group effects to map brain areas with altered connectivity (primary study question) and for group-by-distance interactions to determine whether the alterations concerned to the spatial structure (i.e., differential implication of distinct local distances). In all analyses, results were considered significant when clusters formed at a threshold of *p* < 0.005 survived whole-brain family-wise error (FWE) correction (*p* < 0.05), calculated using SPM.

## Results

The characteristics of the study samples are reported in Table 1.

**Table 1:** Clinical characteristics of the study samples

	Control subjects (*N* = 23)	Osteoarthritis Sample 1 (*N* = 31)	Osteoarthritis Sample 2 (*N* = 34)
Age, years mean	63.3 (7.5)	65.6 (8.8)	63.7 (7.7)
Sex, number F/M	13/10	27/4	26/8
Radiological severity	___	2.0 (0.7)	2.3 (0.5)
WOMAC index, total	3.7 (6.3)	33.0 (11.9)	40.7 (19.6)
PainDetect	2.9 (3.7)	10.5 (5.7)	11.2 (6.2)
Clinical pain intensity (BPI)^a^	4.6 (6.0)	17.4 (5.4)	19.8 (6.7)
Tibial region pain^b^	0.9 (1.3)	7.3 (1.6)	6.9 (2.2)
Number of knee tender points	0.3 (0.7)	3.9 (1.7)	6.7 (2.2)
Temporal summation	0.3 (0.7)	1.8 (1.5)	1.6 (2.6)
Spontaneous body pain (max. 100)	___	___	4 (14)
	Control subjects (*N* = 35)	Fibromyalgia Sample 1 (*N* = 38)	Fibromyalgia Sample 2 (*N* = 63)
Age, years mean	43.9 (6.0)	46.4 (7.5)	49.3 (8.0)
Sex, number F/M	35/0	38/0	63/0
Illness duration, years	___	7.0 (4.6)	6.6 (6.1)
Fibromyalgia tender points, n°	___	16.0 (1.9)	17.1 (1.5)
FIQ, total score	___	66.8 (13.9)	66.6 (13.9)
FIQ, functional capacity	___	4.9 (1.9)	6.0 (2.1)
General Perception of Health-SF36	___	31.2 (18.3)	39.2 (15.2)
HADS ratings for depression	___	8.8 (4.9)	8.9 (3.9)
HADS ratings for anxiety	___	11.3 (4.0)	10.3 (4.9)
Spontaneous body pain (max. 100)	___	71.7 (15.4)	73.1 (16.7)

Quantitative data are expressed as mean (SD)

General Perception of Health according to the 36-Item Short-Form Health Survey (maximum score, 100)

*WOMAC* Western Ontario and McMaster Universities Osteoarthritis Index, *BPI* Brief Pain Inventory, *FIQ* Fibromyalgia Impact Questionnaire (maximum score, 100), *HADS* Hospital Anxiety and Depression Scale

^a^Severity score on items 3 to 6 of the Brief Pain Inventory

^b^Pain generated with a pressure of 4 kg/cm^2^ during 2 s

All study analyses were based on statistical parametric brain maps. In the first analysis, osteoarthritis and fibromyalgia patients were compared with their respective control groups to map brain areas with altered connectivity (primary analysis) and to determine whether the alterations involve the spatial structure (i.e., differential implication of distinct local distances). The ANOVA testing the main effect of the group across the three distances showed significant differences between osteoarthritis patients and control subjects involving the insula bilaterally extending to the ventral-lateral frontal cortex and visual areas in the occipital cortex. Group-by-distance interaction showed a significant effect in the left posterior insula and right anterior insula (Supplementary Table 1 and Supplementary Fig. 1 in Additional file 1).

In fibromyalgia patients, significant differences were identified with their respective controls across the three distances in the sensorimotor cortex involving most levels of the cortical body representation and visual areas in the occipital cortex. Significant group-by-distance interaction was observed in the inferior (opercular) level of the somatosensory cortex, angular gyrus, and visual cortices (Supplementary Table 1 and Supplementary Fig. 1 in Additional file 1).

**Fig. 1:**
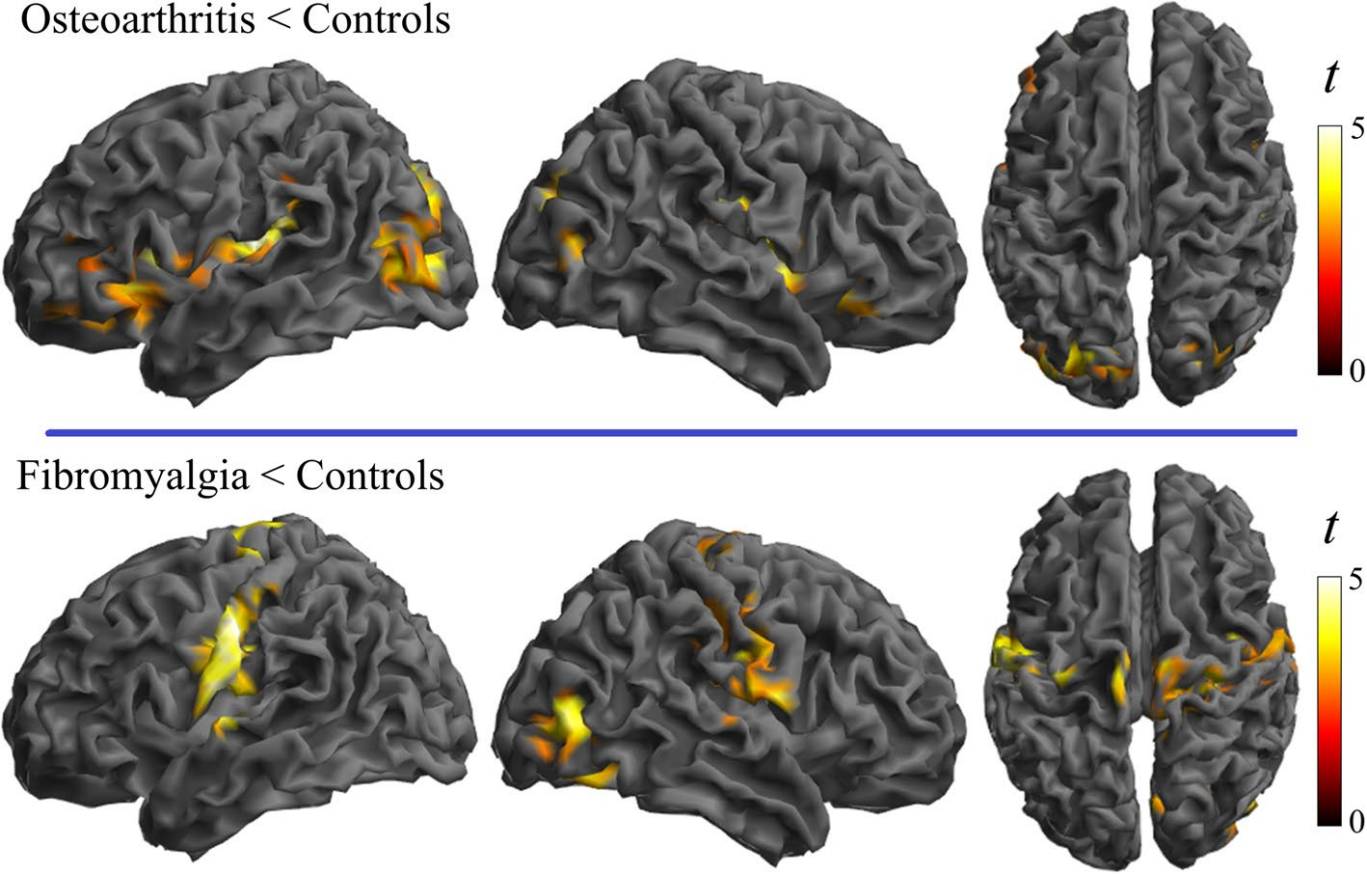
Main effects of group across the three local connectivity distances. Osteoarthritis and fibromyalgia patients are compared with their respective control groups. All the identified alterations were in the direction of weaker connectivity in the patient condition

**Fig. 2:**
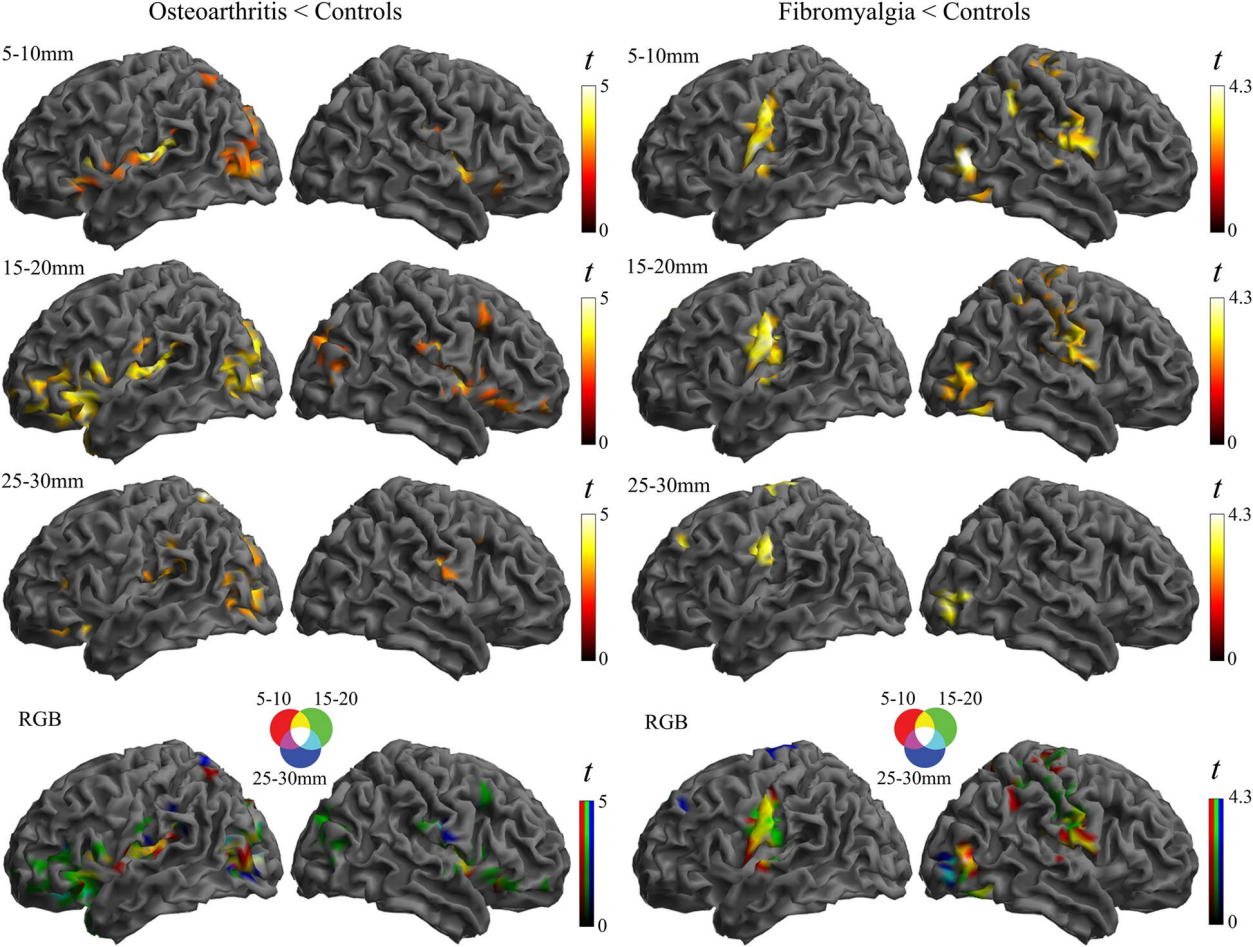
Separate analysis by each local connectivity distance and the RGB composite display. As a general effect, the alteration implicated more the short distances and were all in the direction of weaker connectivity in the patient condition compared with the respective control group

Post hoc analysis comparing patients and control groups showed that all the alterations were in the direction of weaker connectivity in patients compared to control subjects in both osteoarthritis and fibromyalgia (Figs. 1 and 2). The analysis also revealed that, as a general effect, the alteration implicated the short distances more than the long distance, indicating that the dysfunctional effect primarily involves regional, short-distance cortical information transfer (Fig. 2).

**Fig. 3:**
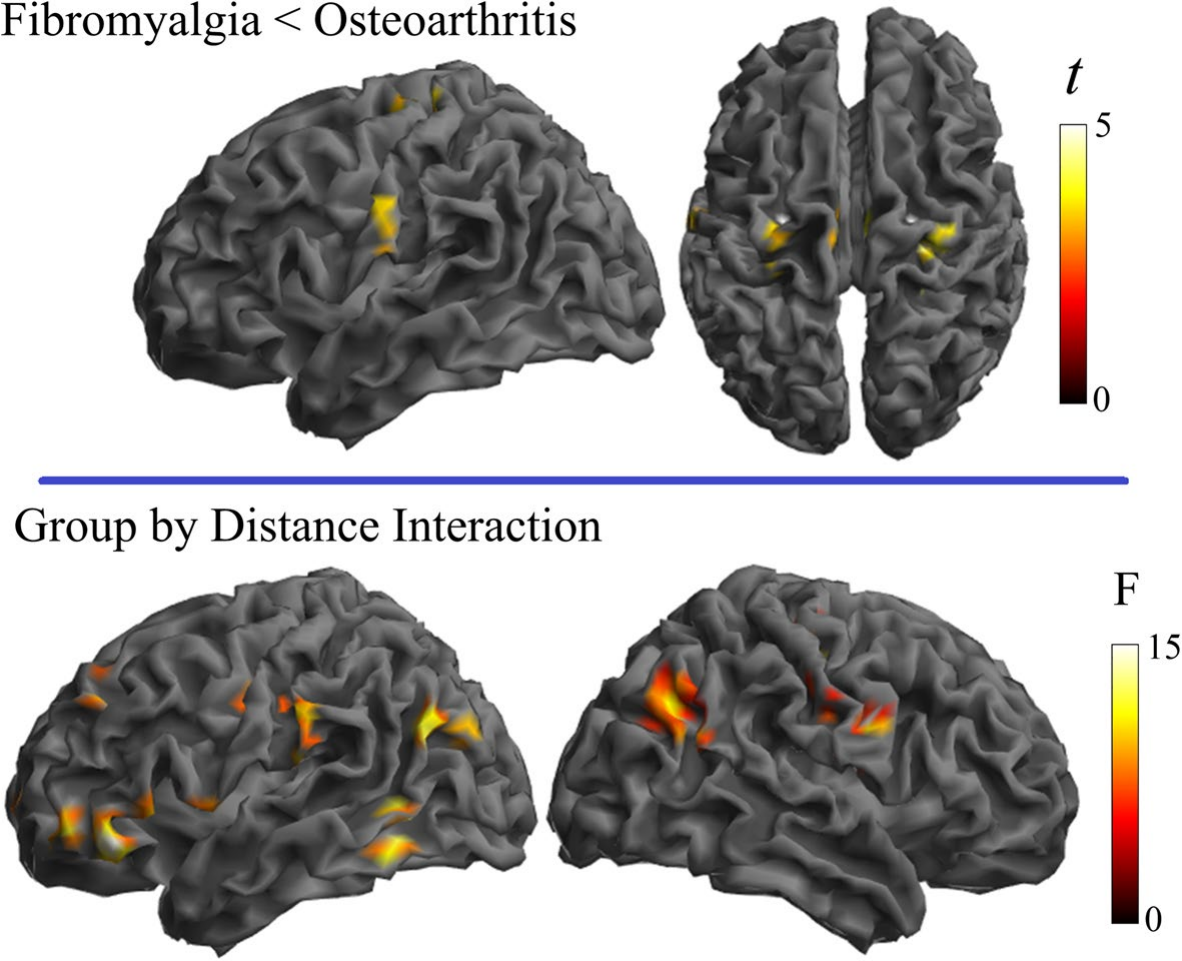
Comparison between osteoarthritis and fibromyalgia patients. Significant main group effect across the three distances (top) and group-by-distance interactions (bottom) are presented

**Fig. 4:**
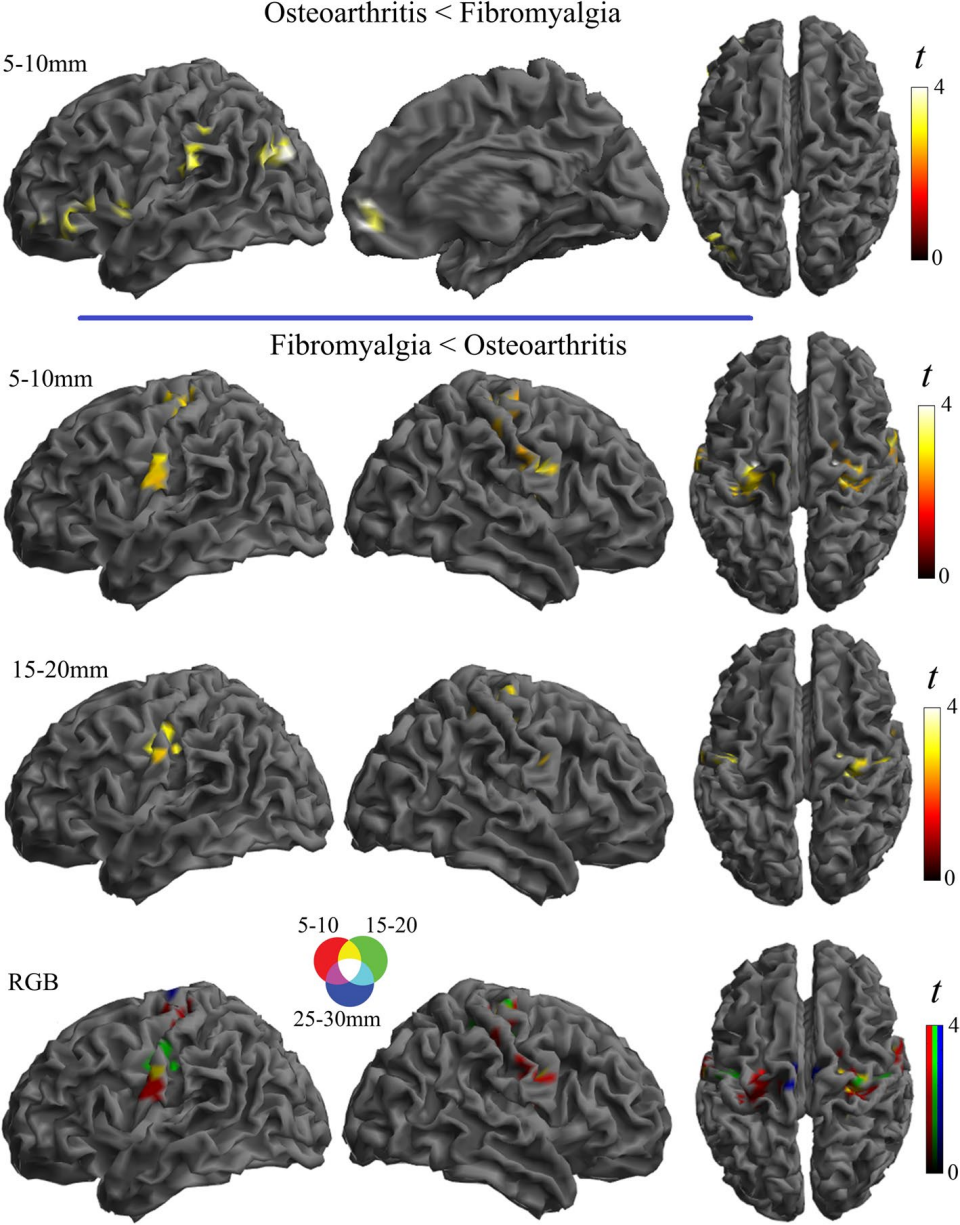
Separate analysis by local connectivity distances comparing osteoarthritis and fibromyalgia patients. The bottom images show the RGB composite display

In the second analysis, a new group of osteoarthritis patients was directly compared with a new group of fibromyalgia patients adjusting for age. Results from group effects across the three distances showed significant differences in the sensorimotor cortex in the direction of weaker connectivity in fibromyalgia patients compared to knee osteoarthritis patients (Supplementary Table 2 in Additional file 1 and Fig. 3). Significant group-by-distance interaction was observed in the inferior (opercular) level of the somatosensory cortex, angular gyri, lateral occipito-temporal junction, and lateral (ventral and dorsal) frontal cortex. A separate analysis by local connectivity distances showed that weaker somatosensory cortex connectivity in fibromyalgia mainly involved the short distances (Fig. 4). In addition, osteoarthritis patients showed weaker connectivity than fibromyalgia patients in the left frontal operculum, inferior parietal lobe, and medial frontal pole. Therefore, patient group comparison overall identified areas overlapping with the patterns of alterations observed when each condition was compared with its respective control group, which confirms that the distribution of cortical dysfunction is distinct in both clinical conditions.

**Fig. 5:**
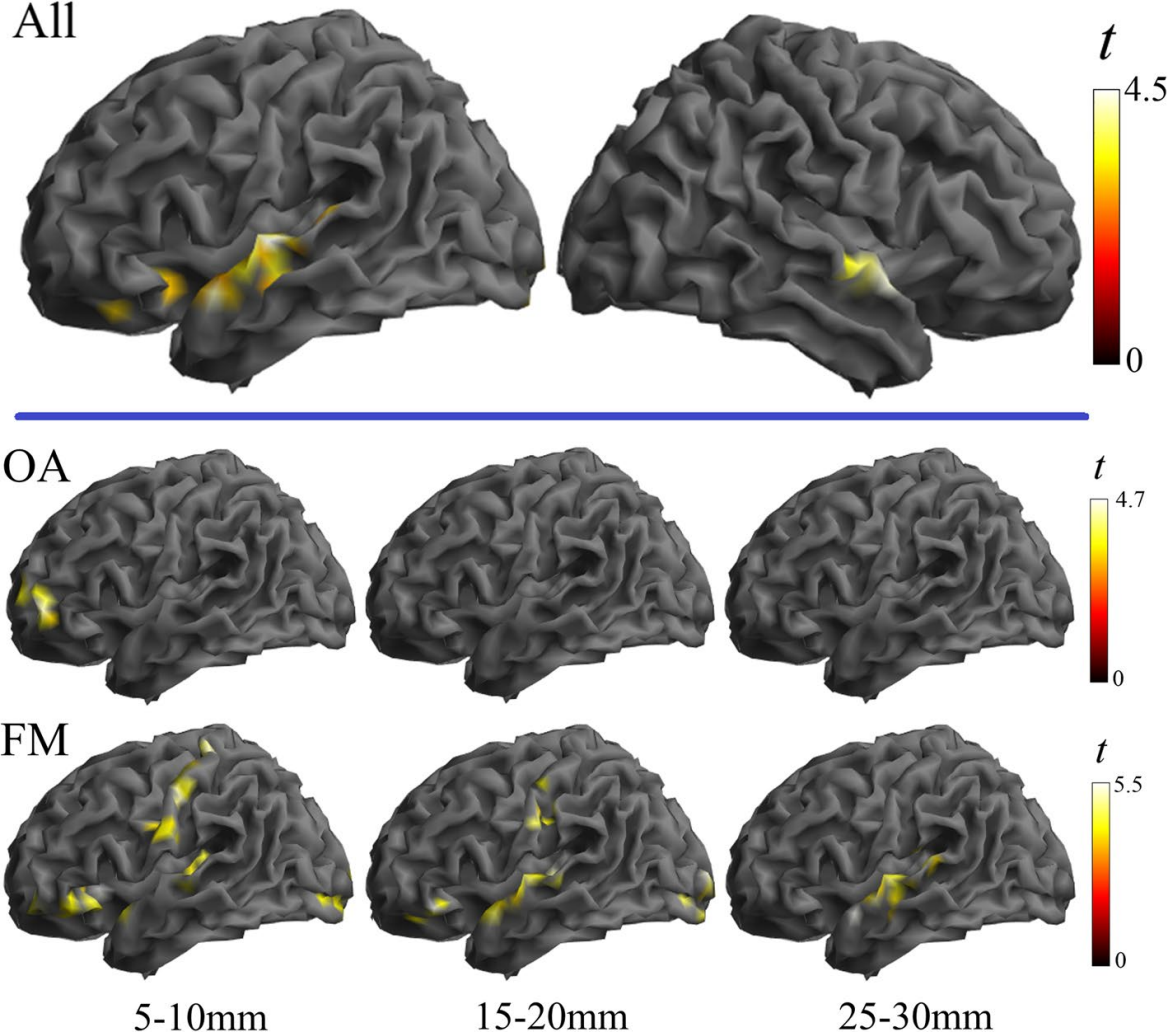
Age correlation analysis results. Top, negative correlation with age (older age, weaker connectivity) across groups and distances, including both osteoarthritis and fibromyalgia patients. The middle and bottom images show significant negative correlations with age in osteoarthritis (OA) and fibromyalgia (FM) patients for each local connectivity distance

A final analysis was conducted to assess the effect of age on study connectivity measures in patients. Across groups and distances, including both osteoarthritis and fibromyalgia patients, the most relevant effect was observed in the auditory cortex showing a negative correlation between functional connectivity and age (Fig. 5, Supplementary Table 3 in Additional file 1). There were no significant results when comparing the age effect between osteoarthritis and fibromyalgia. Separate analyses by group and distances revealed a negative correlation (older age, weaker connectivity) in the auditory cortex, visual cortex, somatosensory cortex, and frontal operculum/anterior insula (gustatory cortex) in fibromyalgia patients. In osteoarthritis patients, a significant negative correlation was restricted to the lateral aspect of the frontal pole at the whole-brain significance threshold. However, a negative correlation with age was observed in the left auditory cortex at a lower threshold (Supplementary Fig. 2 in Additional file 1). This analysis indicates that, although the effect was marginal in osteoarthritis, it was also in the direction of age-related local connectivity weakening in the auditory cortex.

## Discussion

We used combined measures of local connectivity to map functional alterations in the cerebral cortex in two clinical conditions showing augmented sensitivity to pain as a common feature, but with suspected different origins. The results revealed a distinctive pattern of functional alterations in the cerebral cortex. The most relevant alterations in local connectivity for osteoarthritis patients were identified in the insular cortex, which processes important aspects of the brain response to pain [33], whereas fibromyalgia patients showed alterations in the sensorimotor cortex extensively affecting the cortical representation of the body. In both groups, cortical dysfunction occurred in the form of lower connectivity, although this may express distinct pathophysiological alterations in osteoarthritis and fibromyalgia.

Sensitized osteoarthritis patients characteristically show increased pain and neural activity in response to mechanical stimulation [1, 2]. However, in the absence of a stimulus (i.e., at rest), severe knee osteoarthritis is associated with weakened functional connectivity [7, 8] and reduced gray matter volume in the insular cortex [34–36]. Volume reductions in osteoarthritis may be reversible after successful treatment, indicating that the anatomical alteration is not a predisposing factor but rather the consequence of biological stress from long-lasting pain [37–40]. A valuable MR spectroscopy study demonstrated reversible reductions in N-acetylaspartate (NAA), a marker of neural integrity, that led the authors to conclude that augmented neural activity in pain-processing areas would impair mitochondria-dependent energy supply and reduce resting neuronal activity [41]. In this context, lower local connectivity in the insular cortex in our study is compatible with reduced neural activity during metabolic recovery at rest following repeated activation in osteoarthritis patients.

In fibromyalgia patients, imaging research has demonstrated baseline hypometabolism in the brain [42], despite patients complaining of spontaneous, resting pain. Therefore, lower local connectivity in our study is again consistent with reduced overall neural activity in the cortex. In fibromyalgia, however, the situation may be interestingly more intricate. Indeed, neurophysiological studies consistently show a deficient inhibition of the cerebral cortex in fibromyalgia patients [43]. A deficient inhibition from gamma-aminobutyric acid (GABA) interneurons may further contribute to local functional connectivity reduction, due to a loss in their synchronization effect [18]. Therefore, lower intra-regional sensorimotor cortex connectivity may express both reduced activity of principal neurons (fewer active neurons) and reduced activity of inhibitory interneurons (reduced synchronization).

In fibromyalgia, therefore, spontaneous pain and bodily discomfort occur without an obvious sensory input increase in individuals with low basal metabolism in a hyperexcitable sensorimotor cortex. This combination of elements is phenomenologically compatible with a state of deafferentation hypersensitivity, where sensitization is proposed to be the effect of gain enhancement of central sensory reception to compensate a “weak” sensory input [44–47]. “Central gain enhancement,” in the form of over-amplification of multi-level auditory signal reception, has been characterized in the case of hearing loss to account for perceptual distortions in the quiet environment (tinnitus) and loudness intolerance (hyperacusis) [44, 48, 49].

The possibility of a relatively weak sensory input exists in more than one sensory domain in fibromyalgia [50, 51], but let us focus on the proprioceptive system. The female sex is an important risk factor for fibromyalgia [52]. Women characteristically show lower muscle (and tendon) mass relative to the total body mass [53] and thus a less prominent proprioceptive (e.g., muscle spindles and Golgi tendon organs) structure. In addition, a relevant portion (34%) of severe fibromyalgia patients show low levels of insulin-like growth factors [54], which mediate the growth hormone action of stimulating the collagen synthesis in tendon and skeletal muscle and ultimately optimizing their tensile properties [55]. It has been proposed that a primary imbalance between nociceptive/non-nociceptive input, per se, could favor pain perception without the need of a net increase in nociceptive input [20]. In this context, sensory imbalance might also be accentuated from a net increase in body mass. Although obesity is not a necessary condition, a high body mass index (without a parallel increase in muscle mass) is a significant risk for developing fibromyalgia [52, 56, 57]. Interestingly, increases in body mass index were found to be coupled with the expansion of the cerebral cortex representation of the body in children [58], which may well illustrate the somatosensory imbalance in the cortical reception fields.

Beyond the body composition argument, however, the most compelling evidence for a weak proprioceptive input in fibromyalgia is related to its low efficiency. For example, Ehlers-Danlos and related joint hypermobility syndromes are characterized by both high overlap with fibromyalgia symptoms and dysfunctional proprioception [59]. Also, joint hypermobility is three times more frequent in fibromyalgia than in control groups [60]. And, importantly, upon specific proprioception testing in fibromyalgia cohorts, significant alterations have been demonstrated in muscle contraction [61, 62], muscle relaxation [63], vibration perception [61], joint position sense [64–67], and the proprioceptive component of postural control [61, 65, 68–71] with frequent falls [68, 72, 73]. Problems in postural control or balance are also related to altered vestibular and visual dysfunction [68, 73, 74], which may indicate that sensory weakness is not limited to proprioception in fibromyalgia [50, 51].

Therefore, the data support that proprioceptive (non-nociceptive) input may be relatively weak in fibromyalgia. This situation may promote compensatory mechanisms in the form of neural gain enhancement to optimize the signal, albeit at the expense of excessive noise (i.e., pain and sensory discomfort). In the somatosensory system, the phenomenon may be particularly detrimental due to relevant pathway sharing and multi-level crosstalk between mechanical non-nociceptive and nociceptive somatosensory modalities [75, 76], which prevent the amplification of neural signals from being selective. Relevantly, such gain enhancement effects may contribute to low pain tolerance added to the aforementioned primary pain propensity related to nociceptive/non-nociceptive input imbalance itself [20, 75].

Differences in the response to treatments further support distinct pathophysiological mechanisms for pain sensitization in both situations. For example, anti-inflammatory drugs are analgesic in osteoarthritis through the inhibition of nociceptive sensitization [77, 78], whereas opioids, powerful analgesic agents, may paradoxically favor pain sensitization [21], presumably by promoting the release of inflammatory mediators in the nervous system [79]. In fibromyalgia, conventional analgesic/anti-inflammatory drugs are generally not effective [80]. In contrast, significant effects on fibromyalgia symptom relief have been obtained by stimulating proprioception with exercise or instrumental mechanical stimulation [80, 81]. In a broader perspective, research on non-pharmacological treatments for fibromyalgia is expanding with approaches seeking to modify the severity of symptoms through education on the neurobiology of pain, used in combination with exercise [82–84].

A limitation in our study is the comparison of clinical populations typically showing significant age differences. We adopted what we considered to be the optimal strategy to circumvent such a limitation. Each clinical condition was compared with a matched control sample and new samples were used to compare groups adjusting for age. In addition, the effect of age was directly tested in the study samples. We found a general effect of age with local connectivity weakening in the sensory cortex. Importantly, the lowest values in connectivity in the sensory cortex were observed in the young group (i.e., fibromyalgia), which would essentially rule out the effect of age in terms of the differences observed between fibromyalgia and osteoarthritis patients.

We would also mention that the described imaging findings in both pain sensitization conditions do not comprehensively express all the functional repercussions on the brain. We used a specific measure that captured changes in the local functional structure of the cerebral cortex. However, previous research has demonstrated distinct brain alterations using, for instance, large-scale functional connectivity measures [6–16]. It is also relevant to emphasize that fibromyalgia is a complex syndrome showing a variety of comorbid expressions (e.g., migraine, irritable bowel syndrome, chronic fatigue syndrome, depression, and catastrophizing) that were not considered in the present study, whose aim was limited to comparing two clinical disorders.

## Conclusions

In osteoarthritis, we observed functional alterations within the insula at rest, which is a cortical structure showing robust activation during painful stimulation. This finding may be interpreted as an eventual consequence of repeated nociceptive input in this condition. In fibromyalgia, functional disturbances implicated the sensorimotor cortex extensively affecting the cortical representation of the body. In the fibromyalgia neurophysiological context, the identified changes can be better understood in terms of a deafferentation hypersensitivity phenomenon, where sensitization is proposed to be the effect of gain enhancement of central sensory reception to compensate a weak sensory input. We would propose that deficient proprioception could be a contributing factor to weak sensory input. In terms of clinical implications, our study may be relevant to the extent that the results revealed different pathophysiological mechanisms in both clinical conditions. Our observations may support the rational development of therapeutic strategies in pain-sensitized patients, particularly in fibromyalgia by further suggesting the targeting of the proprioceptive system.

## Supplementary Information

**Additional file 1: Supplementary Material.** Description of data: Supplementary Methods. Supplementary Figures. Supplementary Tables.

## Data Availability

The datasets used and/or analyzed during the current study are available from the corresponding author on reasonable request.

## Abbreviations

IDAC: Iso-distance average correlation
NRS: Numerical rating scale
EPI: Echo-planar imaging
MRI: Magnetic resonance imaging
MNI: Montreal Neurological Institute
