# Supplementary material for: Distinctive alterations in the functional anatomy of the cerebral cortex in pain-sensitized osteoarthritis and fibromyalgia patients
Source: Arthritis Res Ther. 2022 Nov 11;24:252. doi: 10.1186/s13075-022-02942-3 (PMC9650905; doi:10.1186/s13075-022-02942-3)
Supplement: Supplementary file 1 — Additional file 1: Supplementary Material. Description of data: Supplementary Methods. Supplementary Figures. Supplementary Tables. [file 13075_2022_2942_MOESM1_ESM.pdf]

Distinctive alterations in the functional anatomy of the cerebral cortex in pain-sensitized osteoarthritis and fibromyalgia patients

Supplementary Material

Supplementary Methods

Supplementary Figures

Supplementary Tables

Supplementary Methods

Iso-Distant Average Correlation (IDAC) maps

A novel mapping was used to characterize the functional structure of the cerebral cortex based on Iso-Distant Average Correlation (IDAC) measures. Essentially, IDAC mapping expands well-established MRI measures of local functional connectivity [6-8] by combining the connectivity maps of varying distances. Composite IDAC maps may uniquely inform the connectivity-related specialization of the cerebral cortex as local connectivity is distance-specific to a large extent and proved to discriminate well between major classical anatomo-functional cortical areas [2,5].

Image processing

Imaging data were processed using MATLAB version 2016a (The MathWorks Inc, Natick, Mass) and Statistical Parametric Mapping software (SPM12; The Wellcome Department of Imaging Neuroscience, London).

Anatomical and functional images were visually inspected to detect possible acquisition artifacts. Functional MRI images were slice-time corrected, realigned and then smoothed by convolving the image with a 4x4x4mm3 full width at half maximum (FWHM) Gaussian kernel.

The resulting realignment parameters were used for scrubbing, namely, discarding motion-affected volumes [3]. For each subject, mean inter-frame motion measurements [4] served as an index of data quality to flag volumes of suspect quality across the run. At time points with mean inter-frame motion > 0.3 mm, the corresponding volume, the immediately preceding and the succeeding two volumes were all discarded. Using this procedure, a mean (± SD) of 3.4 (± 10.2) volumes from the total of 180 volumes included in the fMRI sequence were removed in the Osteoarthritis Sample 1; mean 2.7 (± 6.2) in the corresponding control group; mean 3.6 (± 5.9) in the Fibromyalgia Sample 1; mean 6.2 (± 12.8) in the corresponding control; mean 5.3 (± 10.6) in the Osteoarthritis Sample 2; and mean 0.9 (± 2.5) in the Fibromyalgia Sample 2.

Image volumes were then co-registered to their anatomical images with an affine transformation. A warping matrix was also estimated for every subject to match a group template created from the 3D anatomical individual acquisitions and then to the Montreal Neurological Institute (MNI) space using DARTEL normalization [1]. Image volumes were re-

sliced to 3x3x3 mm. Estimated DARTEL normalizations to the MNI space were applied to the IDAC results to enable group inferences.

IDAC computations (see below) were conducted in a gray matter mask split into left and right hemispheres, so that no adjacent voxels from the medial regions of one hemisphere would be locally associated with those from the other hemisphere. The two hemispheres were brought back together once the IDAC values had been calculated. The left and right hemisphere gray matter masks were obtained by setting a threshold of $p>0.4$ on the gray matter probability maps obtained from the DARTEL group template. As IDAC value estimations were carried out in every subject's native space, the template masks were back-transformed with the inverse estimated normalization.

In IDAC computation analyses, all time series were regressed on the 6 rigid body realignment parameters and their first-order derivatives, and on the average white matter, CSF and global brain signals extracted from the native tissue masks. Finally, all functional MRI time series were band-passed with a Discrete Cosine Transform (DCT) filter letting through frequencies in the 0.01-0.1 Hz interval.

Cases were excluded from initial larger samples on the grounds of imaging quality and the strict mean inter-frame motion [4] criterion of > 0.2 mm: Osteoarthritis Sample 1; 2 cases were excluded from an initial sample of 33. The corresponding control group; 1 from a sample of 24. Fibromyalgia Sample 1; 2 from a sample of 40. Its corresponding control group; 1 from a sample of 36. Osteoarthritis Sample 2; 1 from a sample of 35. Fibromyalgia Sample 2; 2 from a sample of 65.

Whole-cortex IDAC maps were generated by estimating the average temporal correlation of each voxel with all its neighboring voxels placed at increasingly separated Euclidean iso-distant intervals. IDAC was computed in native space separately for each hemisphere after realignment and smoothing. Three IDAC maps were obtained at distance intervals 5-10mm, 15-20mm and 25-30mm.

Multi-distance IDAC color maps were obtained from the overlay of the three IDAC maps using an RGB color codification (see Figures). RGB color channels enabled the display of three values simultaneously. RED corresponding to the results from 5-10mm IDAC map analyses, GREEN from 15-20mm and BLUE from 25-30mm. The overlapping of these primary colors produces a full range of secondary colors.

Definition of Iso-Distant Average Correlation (IDAC)

We defined the concept of "Iso-Distant Average Correlation" (IDAC) to describe the pattern of correlation decay in the close vicinity of a voxel [2]. $IDAC_i(h)$ was consequently defined as the average temporal correlation of voxel *i* with all the voxels located at a given Euclidean distance interval *h*. Functional MRI data sets being a discrete sample, any distance interval h must be necessarily transformed into a discrete iso-distant interval $H_k=(h_k, h_{k+1})$, with h_k being a set of successively increasing distances covering the whole vicinity of a given voxel (see Figure).

The set of iso-distant intervals H_k were selected so that temporal correlations were mainly positive, decreased monotonically and in which horizontal axon collaterals were considered likely to form local networks. For the present study, we defined 3 iso-distant intervals: 5-10mm, 15-20mm and 25-30mm, with constant thicknesses but increasing number of voxels.

We first computed a correlation matrix *C* of Pearson coefficients comparing the functional MRI signal time course of all the voxels in our study mask with each other's.

$$C_{i,j} = \frac{\sum_{k=1}^{M}\left(Y_{i,k} - \overline{Y_i}\right)\cdot\left(Y_{j,k} - \overline{Y_j}\right)}{\sqrt{\sum_{k=1}^{M}\left(Y_{i,k} - \overline{Y_i}\right)^2}\cdot\sqrt{\sum_{k=1}^{M}\left(Y_{j,k} - \overline{Y_j}\right)^2}}$$

where M is the length of the functional MRI signal time series and *i* and *j* index all the voxels entering our study mask. We then transformed the Pearson correlation matrix *C* into a Gaussian distributed z-score correlation matrix Z by applying a Fisher transform.

$$Z_{i,j} = \frac{\sqrt{M-3}}{2}\cdot\ln\left(\frac{1+C_{i,j}}{1-C_{i,j}}\right)$$

We obtained then $IDAC_i(h_k)$ by averaging the correlation coefficients of voxel *i* with all the voxels *j* belonging to the interval H_k.

$$IDAC_i(h_k) = \frac{\sum_{j\epsilon H_{k,i}} Z_{i,j}}{N_{k,i}}$$

In short, IDAC values are defined as the mean correlation z-score between one voxel's functional MRI signal and the functional MRI signal of all the voxels within the iso-distant interval $H_{k,i}$. Note that, for a given distance interval *k*, the number of voxels within the concentric iso-distant interval $N_{k,i}$ is not necessarily the same for every voxel *i* due to the edge effects of the study mask.

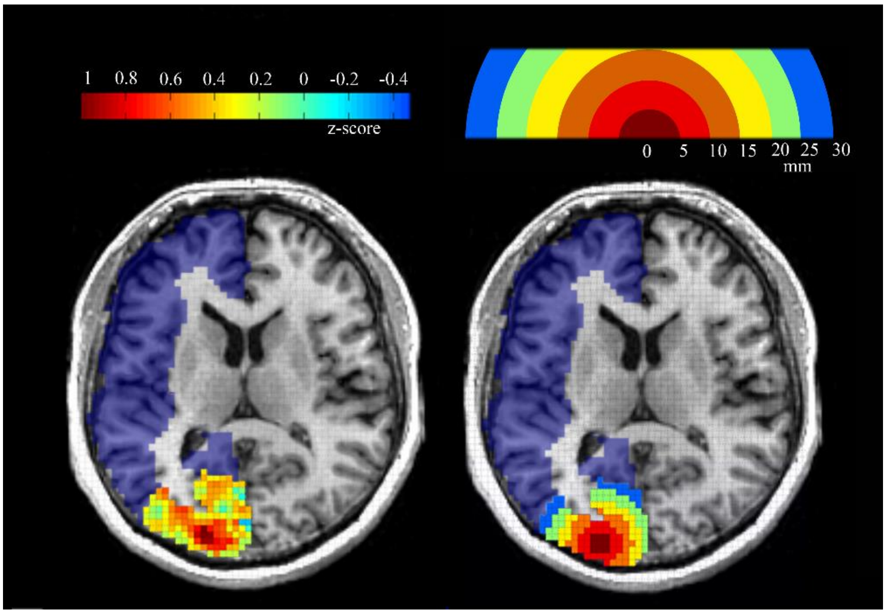

Figure. fMRI Temporal correlations between one voxel ("seed") and its neighboring peripheries present a characteristic decreasing spatial gradient. LEFT: Fisher-transformed z-scores of a correlation map with a "seed" voxel in the visual area from a single subject. Voxel resolution is 3x3x3mm and results are constrained to distance intervals h_k<30mm and within the subject's native gray-matter mask (blue shade). RIGHT: Six different Iso-distant intervals as they are used to calculate different IDAC values in our study.

Supplementary References

1. Ashburner J. A fast diffeomorphic image registration algorithm. Neuroimage. 2007 Oct 15;38(1):95-113.

2. Macià D, Pujol J, Blanco-Hinojo L, Martínez-Vilavella G, Martín-Santos R, Deus J. Characterization of the spatial structure of local functional connectivity using multi-distance average correlation measures. Brain Connect. 2018 Jun;8(5):276-287.

3. Power JD, Mitra A, Laumann TO, Snyder AZ, Schlaggar BL, Petersen SE. Methods to detect, characterize, and remove motion artifact in resting state fMRI. Neuroimage. 2014 Jan 1;84:320-41.

4. Pujol J, Macià D, Blanco-Hinojo L, Martínez-Vilavella G, Sunyer J, de la Torre R, Caixàs A, Martín-Santos R, Deus J, Harrison BJ. Does motion-related brain functional connectivity reflect both artifacts and genuine neural activity? Neuroimage. 2014 Nov 1;101:87-95.

5. Pujol J, Blanco-Hinojo L, Maciá D, Alonso P, Harrison BJ, Martínez-Vilavella G, Deus J, Menchón JM, Cardoner N, Soriano-Mas C. Mapping alterations of the functional structure of the cerebral cortex in obsessive-compulsive disorder. Cereb Cortex. 2019 Dec 17;29(11):4753-4762.

6. Sepulcre J, Liu H, Talukdar T, Martincorena I, Yeo BT, Buckner RL. The organization of local and distant functional connectivity in the human brain. PLoS Comput Biol. 2010;6(6):e1000808.

7. Tomasi D, Volkow ND. Functional connectivity density mapping. Proc Natl Acad Sci U S A. 2010;107(21):9885-9890.

8. Zang Y, Jiang T, Lu Y, He Y, Tian L. Regional homogeneity approach to fMRI data analysis. Neuroimage. 2004;22(1):394-400.

Supplementary Figures

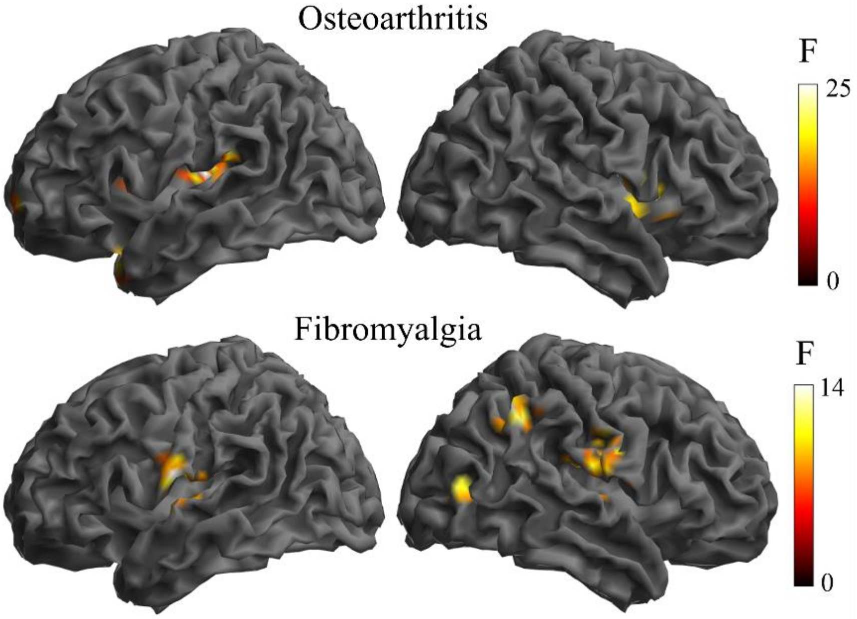

Supplementary Figure 1. Group-by-distance interaction (i.e., group [patients, controls] by distance [5-10 mm, 15-20 mm and 25-30 mm]) significant results in knee osteoarthritis and fibromyalgia patients compared with their respective control groups.

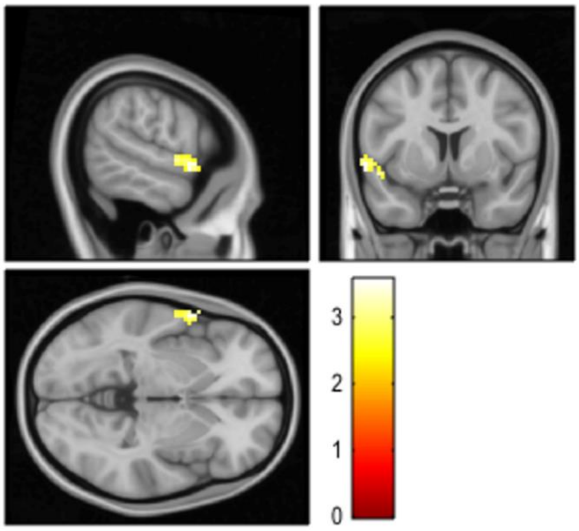

Statistics: *p-values adjusted for search volume*

cluster-level				peak-level					mm mm mm		
$p_{FWE-corr}$	$q_{FDR-corr}$	k_E	p_{uncorr}	$p_{FWE-corr}$	$q_{FDR-corr}$	T	(Z_E)	p_{uncorr}			
0.028	0.019	88	0.000	1.000	0.998	3.57	3.24	0.001	-59	6	-3

Supplementary Figure 2. Negative correlation between age and IDAC values (short distance, 5-10 mm) in knee osteoarthritis patients at a lower threshold (clusters at p< 0.01 surviving whole-brain family-wise error (FWE) correction (p< 0.05)). This analysis indicates that, although the effect was marginal in osteoarthritis, it was also in the direction of age-related local connectivity weakening in auditory cortex in osteoarthritis.

Supplementary Tables

Supplementary Table 1. Functional MRI statistical results. Clinical conditions *versus* the corresponding reference control subject groups

	Cluster-level		*Peak-level*		
	Voxels	$P_{FWE\text{-}corr}$	*x y z*	*F*	*p*
**MAIN EFFECT OF GROUP**					
**Osteoarthritis vs Controls**					
Left Insular Cortex	709	9e-19	-32 -24 15	24.6	2e-6
Left Visual Cortex	333	4e-11	-41 -90 6	24.2	2e-6
Right Insula/Operculum	382	1e-11	49 0 3	27.0	6e-7
Right Visual Cortex	77	0.002	37 -69 6	21.6	7e-6
**Fibromyalgia vs Controls**					
Left Somatosensory Cortex	307	6e-10	-65 -12 39	19.1	0.00002
Left Auditory Cortex	"	"	-68 -15 9	18.6	0.00002
Left Somatosensory Cortex, medial	47	0.04	-11 -27 78	12.3	0.001
Right Somatosensory Cortex, inferior	84	0.002	46 -9 30	22.9	3e-6
Right Somatosensory Cortex, superior	64	0.009	31 -24 42	27.6	4e-7
Right Somatosensory Cortex, medial	66	0.008	7 -15 81	14.7	0.0002
Right Visual Cortex	142	0.00002	49 -84 12	28.8	2e-7
Right Visual Cortex	64	0.009	4 -78 21	21.4	6e-6
**GROUP BY DISTANCE INTERACTION**					
**Osteoarthritis vs Controls**					
Left Insular Cortex	299	2e-11	-38 12 -15	17.6	1e-7
Anterior Cingulate Cortex	51	0.01	4 36 15	14.1	2e-6
Frontal Pole	53	0.008	-20 63 -3	8.8	0.0002
Right Insular Cortex	282	3e-10	46 0 3	25.1	4e-10
**Fibromyalgia vs Controls**					
Left Somatosensory Cortex	196	6e-8	-59 -12 12	11.3	0.00002
Medial Visual Cortex	96	0.0002	1 -84 12	10.5	0.00004
Right Somatosensory Cortex	216	3e-8	52 -15 12	11.7	0.00002
Right Visual Cortex	55	0.01	43 -75 6	13.3	4e-6
Angular Gyrus	88	0.0004	52 -51 45	10.3	0.00006

$P_{FWE\text{-}corr}$, P (Family-Wise Error-corrected), whole brain. x y z, coordinates in Montreal Neurological Institute space.

Supplementary Table 2. Functional MRI statistical results. Osteoarthritis *versus* fibromyalgia

	Cluster-level		*Peak-level*		
	Voxels	$P_{FWE\text{-}corr}$	***x y z***	***t***	***p***
**MAIN EFFECT OF GROUP**					
**Fibromyalgia < Osteoarthritis**					
Left Somatosensory Cortex, medial	54	0.05	1 -15 66	3.7	0.0001
Left Somatosensory Cortex, inferior	66	0.02	-68 -12 21	4.3	0.00001
Left Somatosensory Cortex, superior	63	0.02	-26 -18 48	5.2	2e-7
Right Somatosensory Cortex, superior	116	0.001	31 -21 54	4.6	3e-6
Right Somatosensory Cortex, medial	58	0.04	-2 -9 63	4.2	0.00002
**PATIENT GROUP BY DISTANCE INTERACTION**	***Voxels***	$P_{FWE\text{-}corr}$	***x y z***	***F***	***p***
Left Frontal Operculum	300	4e-11	-35 63 -6	12.8	5e-6
Left Sensorimotor Cortex	45	0.02	-38 -15 30	10.7	0.00003
Left Anterior Medial Frontal Cortex	115	0.00002	-14 69 0	11.7	0.00001
Left Temporo-Occipital Cortex	67	0.002	-62 -63 -12	13.0	4e-6
Left Parieto-Occipital Cortex	119	0.00002	-47 -75 33	10.4	0.00005
Left Dorsolateral Frontal Cortex	60	0.004	-26 39 42	9.3	0.0001
Left Parietal Operculum	61	0.004	-56 -30 15	8.9	0.0002
Left Precuneus	45	0.02	-8 -57 57	8.0	0.0004
Right Somatosensory Cortex, inferior	118	0.00003	52 0 24	16.0	3e-7
Right Inferior Parietal Lobe	277	4e-10	61 -63 27	16.5	2e-7
Right Medial Visual Cortex	169	6e-7	-2 -63 18	14.7	9e-7
Right Sensorimotor Cortex	82	0.001	31 -18 45	13.2	3e-6
Right Medial Frontal Cortex	77	0.001	4 57 0	9.0	0.0002

$P_{FWE\text{-}corr}$, P (Family-Wise Error-corrected), whole brain. x y z, coordinates in Montreal Neurological Institute space.

Supplementary Table 3. Functional MRI statistical results. Age Correlation analysis

	Cluster-level		*Peak-level*		
	Voxels	***$P_{FWE-corr}$***	***x y z***	***t***	***p***
**Osteoarthritis plus Fibromyalgia**					
Left Auditory Cortex	344	3e-9	-59 -18 0	4.5	4e-6
Left Visual Cortex	129	0.0002	1 -102 -9	4.4	0.00001
Left Somatosensory Cortex, medial	58	0.04	4 -18 48	4.1	0.00003
Left Orbitofrontal Cortex	94	0.003	-11 15 -18	3.6	0.0002
Right Auditory Cortex	123	0.001	55 -9 -12	3.6	0.0002
**Osteoarthritis (short distance, 5-10mm)**					
Left Frontal Pole	187	3e-6	-41 51 3	4.7	0.00002
Right Frontal Pole	74	0.009	43 60 -3	4.0	0.0002
**Fibromyalgia (short distance, 5-10mm)**					
Left Auditory Cortex	72	0.005	-59 -18 0	3.8	0.0002
Left Medial Visual Cortex	127	0.00007	1 -102 -9	5.5	4e-7
Left Somatosensory Cortex	75	0.004	-47 -21 51	3.8	0.0001
Left Somatosensory Cortex, opercular	51	0.03	-56 -15 21	3.3	0.001
Left Insula	118	0.0001	-35 21 -3	4.5	0.00002
Left Orbitofrontal Cortex	157	9e-6	-35 54 -15	4.7	0.00001
Right Somatosensory Cortex	131	0.0001	43 -21 51	4.4	0.00002
Right Visual Cortex	290	1e-8	22 -105 -6	5.4	5e-7
Right Insula	102	0.0007	46 18 -9	4.5	0.00002
Right Prefrontal Cortex	56	0.03	55 18 24	3.9	0.0001

$P_{FWE-corr}$, P (Family-Wise Error-corrected), whole brain. x y z, coordinates in Montreal Neurological Institute space.
